# Psychotherapy Training in a Competency-Based Medical Education Psychiatry Residency: A Proposal for a Practical and Socially Responsible Model

**DOI:** 10.1177/07067437211063096

**Published:** 2021-12-22

**Authors:** Mark Fefergrad, Benoit H. Mulsant

**Affiliations:** 1 Department of Psychiatry, 12366 University of Toronto, Toronto, Ontario, Canada; 2 Sunnybrook Health Science Centre, Toronto, Ontario, Canada; 3 Centre for Addiction and Mental Health, Toronto, Ontario, Canada

**Keywords:** psychotherapy, education, training, psychiatry, competency-based medical education

In many jurisdictions, the transition from a time-based to a competency-based model of medical education (CBME) requires reassessing curricula and mandatory training experiences. In Canada, the national roll-out of this new model for psychiatry residencies in 2020 has highlighted challenges related to the nature and scope of the discipline. In particular, current views diverge about the role of psychotherapy in the future of psychiatry. Some argue psychotherapy differentiates psychiatry from other medical specialties because psychiatry and psychotherapy practice remain inseparably linked. Others argue contemporary psychiatry should be based on a neurobiological understanding of mental disorders and psychotherapy should be provided by allied mental health professionals.^1,2^ In an influential survey, psychiatrists and psychiatry educators ascribed less importance to long-term and social psychotherapies, and more importance to descriptive or biological psychiatry.^3^ These 2 views have divergent implications for training. In this Perspective, we describe a middle ground and propose a training model that recognizes that most psychotherapy services and most psychotherapy research are now performed by non-physicians^4^ but emphasizes that all psychiatrists should continue to learn and use psychotherapy skills.

## Psychotherapies are Evidence-Based Interventions Recommended for Most Mental Disorders

Many psychotherapy modalities have a strong evidence base and, alone or in combination with pharmacotherapy constitute core interventions for most mental disorders. For example, the CANMAT guidelines endorse the use of cognitive behavioral therapy (CBT) as a first-line intervention for both the acute treatment and relapse prevention of major depressive disorder (MDD).^5^ This recommendation is based on multiple randomized controlled trials and. meta-analyses.^6^ Similar evidence exists both for other mental conditions – for example, schizophrenia,^7^ borderline personality disorders^8^- and other psychotherapy modalities – such as, psychodynamic therapy,^9^ interpersonal therapy,^10^ or dialectic behavioral therapy.^11^

## Lack of Access to Psychotherapy and its Implication for Residency Training

The Mental Health Commission of Canada (MHCC) reports that only a minority of Canadian adults and children with mental health concerns have access to services or professional support.^12^ They recommend a wider and more equitable access ‘*to psychotherapies and clinical counselling by service providers who are qualified to deliver approaches that are based on best available evidence*’ (p. 4).^12^ Similarly, in Quebec, the Commissioner for Health and Well-Being states, ‘*given the numerous arguments in favour of psychotherapy, the issue facing Canadian policy-makers is no longer whether to increase access to it, but rather to consider what is the best approach to providing broader and more equitable access to psychotherapy services.*’ (p. 101)^13^ In the Canadian publicly funded health system, these calls to improve access to psychotherapy and the question of who should be compensated to provide it,^14^ raise 2 related questions about psychotherapy training in the new CBME psychiatry residency: what psychotherapy competencies should be graduating psychiatry residents achieve? How should the training be delivered?

Given the lack of access to psychotherapy,^15,16^ we do not endorse the position that psychiatrists should be prohibited from providing psychotherapy. However, we also do not believe that psychiatry residency training should aim to produce full-time psychotherapists. Data from Ontario suggest that most psychiatrists who exclusively practice psychotherapy are located in the 2 largest cities, Toronto or Ottawa, and they treat over long periods of time a small number of patients who typically are not severely ill and are economically advantaged.^15^ A small number of psychiatry residents still use most of their selective and elective time to improve their psychotherapeutic skills; they may ultimately decide to focus their practice solely on psychotherapy. We argue, for the reasons of economics and patient access to care, that they should also focus on treating patients with severe mental illness who are not treated by other mental health professionals (e.g., patients who are acutely suicidal, psychotic, or with complex physical co-morbidities).

## What Psychotherapy Competencies Should Graduating Psychiatry Residents Achieve?

In the context discussed above, we propose that psychotherapy core skills and competencies required from all psychiatry residents should meet 2 criteria: ‘need’ (from a population burden of illness perspective) and ‘applicability’ (meaning the skills can be applied in multiple settings). The University of Toronto has developed a list of psychotherapy competencies that meet these 2 criteria and are observable (available upon request). The feasibility of this approach in CBT and psychodynamic therapy has already been evaluated.^17^ This approach can be applied to psychotherapy training more broadly. For example, given the ubiquity of sleep disturbances in patients with mental illness,^18^ CBTi skills are fundamental to the specialty. Similarly, given the co-morbidity of mental illness and addiction,^19^ all psychiatrists need to acquire motivational interviewing skills.

## How Should Psychotherapy Training be Delivered to Psychiatry Residents?

Residency training in any medical specialty is constrained by limitations of time and other resources (e.g., availability of supervisors). Training also needs to rely on fundamental educational principles and to conform to the accreditation requirements associated with CBME (e.g., observable competencies that can be achieved in a sequential manner). Finally, any proposed model of training should be practical so it can be implemented in the 17 accredited psychiatry residency programs in Canada. In this context, among the infinite number of possible psychotherapy training models, we discuss the relative merits of 3 exemplars: (i) the ‘traditional’ model of the Canadian Royal College that had guided training in Canada until 2020; (ii) a CBME ‘familiarity’ model from the American Association of Directors of Psychiatry Residency Training (AADPRT) in the United States (US); (iii) a new ‘applied’ model that we propose as a viable alternative to address some of the limitations and challenges of the 2 other models (Table 1). We acknowledge that these are convenient models to contrast, but also that there is an infinite number of options or hybrids that could be considered.

**Table 1.** Comparison of 3 Psychotherapy Training Models.

	Traditional Model of the Canadian Royal College	Familiarity Model of the American Association of Directors of Psychiatry Residency Training	Proposed Applied Model
Requires exposure to theory	++	+	+
Requires ‘pure’ experiential learning	+++	+	++
Application of psychotherapy skills to ‘non-psychotherapy patients’	−	−	+++
Opportunity for additional exposure to didactic or experiential learning during elective time	+	++	+++
Flexibility for learners	−	++	+++
Requires supervisory resources	+++	+	++

+++: strong emphasis; ++: medium emphasis; +: low emphasis; −: not included.

### Traditional Model

During the past 2 decades, the Canadian Royal College required psychiatry residents to achieve introductory knowledge, working knowledge or proficiency in various psychotherapeutic modalities as^20^ defined by:

– *Introductory knowledge: Able to recognize, identify, or, describe principles*.
– *Working knowledge: Able to demonstrate core aspects of Psychiatry, such as basic interviewing, problem formulation and treatment. The resident can understand the scientific literature*.
– *Proficient: Able to demonstrate working knowledge enhanced by a developmental, cultural, and lifespan perspective, allowing detailed interviewing and bio-psychosocial problem formulation with capacity to teach, consult, assess and manage referrals. The resident can critically review and apply the scientific literature relevant to this competency* (p. 3).^20^

These requirements were associated with specific time-based experiences, which for psychotherapy were, ‘*no less than thirty-two (32) weeks or eight (8) months of the PGY [post-graduate year] 2–5 experience […] In addition to seminars or structured learning activities which are sufficient for basic knowledge, working knowledge is attained by the resident participating as an observer or co-therapist while proficiency is attained by the resident acting as the primary therapist and engaging in supervision one (1) hour per week*.’ (p. 4)^20^

In addition, the Royal College required that residents demonstrate ‘*proficiency in the delivery of cognitive behavioural therapy (CBT), family or group therapy (and working knowledge in the other), psychodynamic therapy and supportive therapy […] working knowledge in the delivery of behavioural therapy, dialectical behavior therapy (DBT), family or group therapy (with proficiency in the other) and interpersonal therapies (IPT)’. Finally, introductory knowledge was ‘required for brief psychodynamic psychotherapy, mindfulness training, motivational interviewing (MI) and relaxation’* (p. 6).^21^

This traditional model required a broad range of experiences and exposed residents to multiple evidence-based psychotherapy modalities. However, its extensive requirements took up a minimum of one-sixth (17%) of training time in the PGY2–5 years which necessarily took time away from other areas of need in the field. Further, their completion required that residents engage in specific psychotherapy modalities with a series of identified patients and individual supervision for each hour of psychotherapy delivered. Based on our observation of more than 500 residents over more than a decade, we believe that typical psychiatry residents who meet these requirements do not have enough experience to become skilled (‘proficient’) psychotherapists by the time they complete their residency. Residents wanting to practice specific psychotherapy have to complete supplementary training involving additional didactic learning and treatment of patients under supervision. Furthermore, some residents are resentful because they perceive that their professional interests and this training in several psychotherapy modalities are not aligned. They see these requirements as onerous and potentially detracting from their career goals.

### Familiarity Model

In 2018, in the context of the implementation of CBME in psychiatry residency in the US, a task force of the US AADPRT published proposed Entrustable Professional Activities (EPA) psychiatry residents should achieve at the end of their training.^22^ Using a Delphi model, this task force determined that CBT and psychodynamic psychotherapy did not meet their criterion for inclusion as an EPA, which consisted of a content validity index of at least 0.8 based on the proportion of respondent directors who rated an item as ‘high’ or ‘very high’ on an essentialness score. An asymmetric confidence interval associated with each mean essentialness rating was also calculated to protect against ‘*the artificial narrowing of the confidence interval that can occur with skewed data’* (p. 1050).^22^ The task force noted that there is ‘agreement that all residents should have exposure to these [psychotherapy] practices and certainly know when to refer a patient to these modalities […] the disagreement centers on whether all residents should obtain ‘competence for independent practice’ by the end of training or whether graduation might require a lower threshold such as entrustment for indirect supervision’ (p. 1053).^22^

In part, this result may be due to the current scope of the profession and context in the US where the provision of psychotherapy by psychiatrists has been on the decline.^23^ This change coincides with changes in reimbursement and increases in managed care where most insurance cover psychotherapy at a rate applicable to all qualified mental health professionals.

While this familiarity model is efficient with respect to training time, it emphasizes a theoretical understanding of various psychotherapeutic modalities instead of experiential learning of these modalities with supervision. By design, most graduating residents are not expected to be ready confident enough to provide psychotherapy independently. Furthermore, without experiential training, many graduating residents will not be able to integrate specific psychotherapy skills into the provision of psychiatric care. Data suggest that didactic knowledge about psychotherapy and other similar skills (e.g., communication or teaching) is not sufficient to be able to use them.^24^ Novice psychotherapists benefit from supervision of ongoing cases that provides them with a deeper understanding of the therapeutic relationship, how to manage their own reactions, and act therapeutically under challenging circumstances.^25^

### Applied Model

Given the challenge related to access to psychotherapy provided by psychiatrists and its implication for psychiatry training, we propose a model focused on teaching ‘psychotherapy skills’. This model includes the didactic teaching embedded in the other 2 models; it also provides some early exposure to several ‘pure’ psychotherapeutic modalities. However, unlike the traditional Royal College model that encourages broad but shallow exposure to multiple modalities, this model emphasizes the application of psychotherapy skills to multiple patient populations and settings.

The design of this model is based on several informed assumptions. First, the majority of graduates from Canadian psychiatry residencies will not provide traditional individual psychotherapy independent of other interventions (e.g., pharmacotherapy). Second, psychotherapy remains an essential intervention to improve both acute and long-term outcomes in most patients with severe mental illness^26^ because all psychiatric interventions can be enhanced by the application of psychotherapeutic principles to improve engagement and adherence.^27^ However, while psychotherapy delivered by a physician is covered ‘free of charge’ by most provincial health plans in Canada, most Canadian patients who could benefit from psychotherapy do not have access to it because there are not enough psychiatrists or other qualified physicians to deliver psychotherapy.^28^ Given the prevalence of mental illness, it is not possible to train enough physicians to meet the need for psychotherapy.^29^ Our final, assumption is that psychotherapy skills can have an impact on patient outcomes. While we acknowledge that this crucial assumption has not been directly tested in a randomized clinical trial, it is supported by evidence from computer-based interventions, ultra-short interventions, and other indirect evidence such as the impact of manual-based psychotherapy provided by lay providers.^30^

Thus, we propose a training model aiming to generate competence in the use of a variety of ‘psychotherapy skills’ rather than competence in the delivery of a specific formal course of psychotherapy. For example, using the techniques of Socratic questioning and thought records inherent to CBT may help outpatients to identify and modify their dysfunctional thoughts around medication adherence. Similarly, DBT skills may help a patient presenting with dysregulated affect in an emergency department. Psychodynamic skills may be of value during family meetings or to engage inpatients who are reluctant to talk to a psychiatrist. In this model, as in the AADPRT familiarity model, psychiatry residents acquire didactic and experiential familiarity with a small number of psychotherapy modalities. They also learn to apply psychotherapy skills that are useful to most patients in most settings. These skills have been defined by both national and local expert consensus. Consistent with the apprenticeship associated with CBME, the acquisition of these skills is ensured through a combination of direct supervision (i.e., being in the room, or using audio/video recordings) and indirect supervision (i.e., resident report, review of clinical notes) integrated into regular psychiatric care. This model provides the additional advantage of necessitating a discussion with supervisors about which skills to use with each patient in a given context (i.e., specific therapeutic skills targeting specific symptoms or behaviors in a specific patient seen in a specific setting). These discussions were largely moot in the traditional model as a predetermined psychotherapy was delivered to a small number of patients pre-selected for the lack of co-morbid or characterological complexity. We acknowledge that this model could potentially have some disadvantages. For example, if a very small number of residents opted to pursue additional training in psychotherapy, access could be diminished. Another potential challenge is the need for increased faculty development so that residents can be supported in using psychotherapy in clinical situations and settings that are less suitable for psychotherapy than in the traditional model.

The observation of skills proposed in our model is entirely compatible with the Royal College's CBME program which all 17 schools are currently implementing. This would support a smooth implementation across the country if adopted. However, each program and ultimately each resident could customize their training beyond the basic requirements outlined above. While most residents would have fewer psychotherapy training hours, the early experiential training in specific psychotherapy modalities would continue to identify the subset of psychiatry residents who decide to pursue additional training in these modalities. Residents interested in developing advanced expertise in psychotherapy would have access to more institutional resources. In turn, more psychiatrists could conduct academic work (i.e., teaching and research) in medical psychotherapy. Reducing the number of required core modalities increases the available elective time to deepen psychotherapeutic skills, train in additional elective modalities, or develop expertise in other areas. Like current models of sub-specialty training, this model would result in solid core competencies for general psychiatrists, while allowing for the development of true expertise in a subset of self-selected residents.

## References

[1] Doidge N . Opinion: In Ontario, a battle for the soul of psychiatry. Retrieved October 16, 2020. 2019. https://www.theglobeandmail.com/opinion/article-in-ontario-a-battle-for-the-soul-of-psychiatry/

[2] Paris J . Is psychoanalysis still relevant to psychiatry? Can J Psychiatry. 2017;62(5):308-312. doi: 10.1177/0706743717692306 28141952 PMC5459228

[3] Langsley DG Yager J . The definition of a psychiatrist: eight years later. Am J Psychiatry. 1988;145(4):469-475. doi: 10.1176/ajp.145.4.469 3348450

[4] TALK AND TRANSFORMATION – eopa.ca. (n.d.). Retrieved October 16, 2020. https://eopa.ca/sites/default/uploads/files/Talk and Transformation_Recommendations for a structured psychotherapy program in Ontario(1).pdf

[5] Parikh SV Quilty LC Ravitz P et al. 2018. Canadian Network for Mood and Anxiety Treatments (CANMAT) 2016 Canadian Network for Mood and Anxiety Treatments (CANMAT) 2016 Clinical Guidelines for the Management of Adults With Major Depressive Disorder: section 2. Psychological treatments. Can J Psychiatry. 2016;61(9):524-539. 10.1177/0706743716659418 PMC4994791 27486150

[6] Cuijpers P Berking M Andersson G , et al. A meta-analysis of cognitive-behavioural therapy for adult depression, alone and in comparison with other treatments. Can J Psychiatry. 2013;58(7):376-385. doi: 10.1177/070674371305800702 23870719

[7] Norman R Lecomte T Addington D Anderson E . Canadian treatment guidelines on psychosocial treatment of schizophrenia in adults. Can J Psychiatry. 2017;62(9):617-623. doi: 10.1177/0706743717719894 28703017 PMC5593243

[8] Jorgensen MS Storebo OJ Simonsen E . Systematic review and meta-analyses of psychotherapies for adolescents with subclinical and borderline personality disorder: methodological issues. Can J Psychiatry. 2020;65(1):59-60. doi: 10.1177/0706743719893893 31813274 PMC6966256

[9] Fonagy P . The effectiveness of psychodynamic psychotherapies: an update. World Psychiatry. 2015;14(2):137-150. doi: 10.1002/wps.20235 26043322 PMC4471961

[10] Cuijpers P Donker T Weissman MM , et al. Interpersonal psychotherapy for mental health problems: a comprehensive meta-analysis. Am J Psychiatry. 2016;173(7):680-687. doi: 10.1176/appi.ajp.2015.15091141 27032627

[11] DeCou CR Comtois KA Landes SJ . Dialectical behavior therapy is effective for the treatment of suicidal behavior: a meta-analysis. Behav Ther. 2019;50(1):60-72. doi: 10.1016/j.beth.2018.03.009 30661567

[12] Mental Health Commission of Canada. Changing directions, changing lives: The mental health strategy for Canada. Calgary, AB. 2012.

[13] Commissaire à la santé et au bien-être. Rapport d’appréciation de la performance du système de santé et de services sociaux : pour plus d’équité et de résultats en santé mentale au Québec. Quebec City, Quebec, Canada: Gouvernement du Québec; 2012.

[14] Expanding Access to Psychotherapy: Mapping Lessons Learned. Retrieved October 16, 2020. 2018. https://www.mentalhealthcommission.ca/sites/default/files/2018-08/Expanding_Access_to_Psychotherapy_2018.pdf

[15] Rudoler D De Oliveira C Zaheer J , et al. Closed for business? Using a mixture model to explore the supply of psychiatric care for new patients. Can J Psychiatry. 2019;64(8):568-576. doi: 10.1177/0706743719828963 30803265 PMC6681508

[16] Bartram M . Expanding access to psychotherapy in Canada: building on achievements in Australia and the United Kingdom. Healthc Manage Forum. 2019;32(2):63-67. doi: 10.1177/0840470418818581 30700162

[17] Ravitz P Lawson A Fefergrad M , et al. Psychotherapy competency milestones: an exploratory pilot of CBT and psychodynamic psychotherapy skills acquisition in junior psychiatry residents. Acad Psychiatry. 2018;43(1):61-66. doi: 10.1007/s40596-018-0940-4 29858773

[18] Ohayon MM Guilleminault C . Epidemiology of sleep disorders. Sleep Disord Med. 2009;10(9): 952-960. doi: 10.1016/b978-0-7506-7584-0.00021-5

[19] Veldhuizen S Urbanoski K Cairney J . Geographical variation in the prevalence of problematic substance use in Canada. Can J Psychiatry. 2007;52(7):426-433. doi: 10.1177/070674370705200704 17688006

[20] Specialty Training Requirements in Psychiatry. (n.d.). Retrieved October 16, 2020. http://www.royalcollege.ca/rcsite/documents/ibd/psychiatry_str_e

[21] Objectives of Training in the Specialty of Psychiatry. (n.d.). Retrieved October 16, 2020. http://www.royalcollege.ca/rcsite/documents/ibd/psychiatry_otr_e

[22] Young JQ Hasser C Hung EK , et al. Developing end-of-training entrustable professional activities for psychiatry. Acad Med. 2018;93(7):1048-1054. doi: 10.1097/acm.0000000000002058 29166349

[23] Mojtabai R Olfson M . National trends in psychotherapy by office-based psychiatrists. Arch Gen Psychiatry. 2008;65(8):962. doi: 10.1001/archpsyc.65.8.962 18678801

[24] Napel-Schutz MCT Abma TA Bamelis LLM , et al. How to train experienced therapists in a new method: a qualitative study into therapists views. Clin Psychol Psychother. 2016;24(2):359-372. doi: 10.1002/cpp.2004 26791440

[25] Strupp HH . The Vanderbilt psychotherapy studies: synopsis. J Consult Clin Psychol. 1993;61(3):431-433. doi: 10.1037//0022-006x.61.3.431 8326043

[26] Koelen J Houtveen J Abbass A , et al. Effectiveness of psychotherapy for severe somatoform disorder: meta-analysis. Br J Psychiatry. 2014;204(1):12-19. doi: 10.1192/bjp.bp.112.121830 24385460

[27] Day JC Bentall RP Roberts C , et al. Attitudes toward antipsychotic medication: the impact of clinical variables and relationships with health professionals. Arch Gen Psychiatry. 2005;62(7):717-724. 15997012 10.1001/archpsyc.62.7.717

[28] Alvine Fansi A Jehanno C . Avis sur l’accès équitable aux services de psychothérapie. Quebec City, QC: INESSS; 2015.

[29] Kurdyak P Zaheer J Carvalho A , et al. Physician-based availability of psychotherapy in Ontario: a population-based retrospective cohort study. CMAJ Open. 2020;8(1):E105-E115. doi: 10.9778/cmajo.20190094 PMC7065559 32161044

[30] Hedman E Ljotsson B Kaldo V , et al. Effectiveness of internet-based cognitive behavior therapy for depression in routine psychiatric care. J Affect Disord. 2014;55:49-58. 10.1016/j.jad.2013.10.023 24238951

